# Optimization of Nanowell-Based Label-Free Impedance Biosensor Based on Different Nanowell Structures

**DOI:** 10.3390/bios14090426

**Published:** 2024-09-04

**Authors:** Ali Fardoost, Hassan Raji, Mehdi Javanmard

**Affiliations:** Department of Electrical and Computer Engineering, Rutgers University, Piscataway, NJ 08854, USA; af1069@scarletmail.rutgers.edu (A.F.); hr322@soe.rutgers.edu (H.R.)

**Keywords:** label-free biosensor, IL-6 protein, impedance-based biosensor, cancer biomarker, nanowells, sensitivity

## Abstract

Nanowell-based impedance-based label-free biosensors have demonstrated significant advantages in sensitivity, simplicity, and accuracy for detecting cancer biomarkers and macromolecules compared to conventional impedance-based biosensors. Although nanowell arrays have previously been employed for biomarker detection, a notable limitation exists in the photolithography step of their fabrication process, leading to a reduced efficiency rate. Historically, the diameter of these nanowells has been 2 μm. To address this issue, we propose alternative geometries for nanowells that feature larger surface areas while maintaining a similar circumference, thereby enhancing the fabrication efficiency of the biosensors. We investigated three geometries: tube, spiral, and quatrefoil. Impedance measurements of the samples were conducted at 10 min intervals using a lock-in amplifier. The study utilized interleukin-6 (IL-6) antibodies and antigens/proteins at a concentration of 100 nM as the target macromolecules. The results indicated that tube-shaped nanowells exhibited the highest sensitivity for detecting IL-6 protein, with an impedance change of 9.55%. In contrast, the spiral, quatrefoil, and circle geometries showed impedance changes of 0.91%, 0.95%, and 1.62%, respectively. Therefore, the tube-shaped nanowell structure presents a promising alternative to conventional nanowell arrays for future studies, potentially enhancing the efficiency and sensitivity of biosensor fabrication.

## 1. Introduction

In the area of biosensing technologies, label-free biosensors have emerged as indispensable tools, fundamentally reshaping the way we detect and analyze biomolecular interactions with unprecedented precision and efficiency. Unlike label-based methods such as chemiluminescence, enzyme-linked immunosorbent assay (ELISA), fluorescence-based assays, and colorimetric assays, which often entail time-consuming processes, intricate labeling steps, and susceptibility to signal interference and variability, label-free biosensors harness intrinsic properties of target molecules, such as electrical impedance, atomic mass, and size [1,2,3,4,5]. Consequently, they offer a plethora of advantages, including simplicity, real-time monitoring, flexibility, sensitivity, specificity, cost-effectiveness, and seamless integration, positioning them as a superior alternative to label-based counterparts.

Within the domain of label-free biosensors, electrical biosensors, particularly impedance-based ones, have emerged as a compelling option for detecting biomarkers and facilitating diagnostic applications in the medical field, owing to their affordability, energy efficiency, and scalability. These impedance-based biosensors operate on the principle of detecting changes in impedance at the electrode–solution interface upon encountering the target molecule [6,7]. Also, they facilitate the binding of antibodies to the electrode’s surface, offering a robust foundation for detecting the presence of both antigens captured by the antibodies [8]. Nevertheless, aggregation, unintended bindings, and clustering of antibodies on the electrode surface can generate undesired captured signals, potentially resulting in detection errors and compromising the sensitivity of the sensor [9].

It has been revealed that nanowell-based biosensors exhibit significant potential for delivering remarkably sensitive label-free detection by preventing nonspecific bindings, all without the need for chemical or biological reagents. Additionally, prior research indicates that the electrodes in nanowell-based biosensors offer the advantage of swiftly detecting biomolecules with enhanced reproducibility, owing to their capability to alleviate mass transfer constraints [9,10,11,12,13]. Thus, nanowell-based biosensors offer a solution to the previously mentioned drawbacks of impedance-based biosensors.

In the context of this study, interleukin-6 (IL-6) serves as the target macromolecule. Leveraging the highly selective interaction between antigen and antibody, IL-6 proves to be an optimal choice for immunosensing applications [14,15]. IL-6, a multifunctional cytokine, plays a crucial role in inflammation, immune regulation, hematopoiesis, metabolism, neurology, and cancer biology. Its dysregulation has been linked to various diseases, underscoring its significance as a target for therapeutic interventions [16,17].

**Figure 1:**
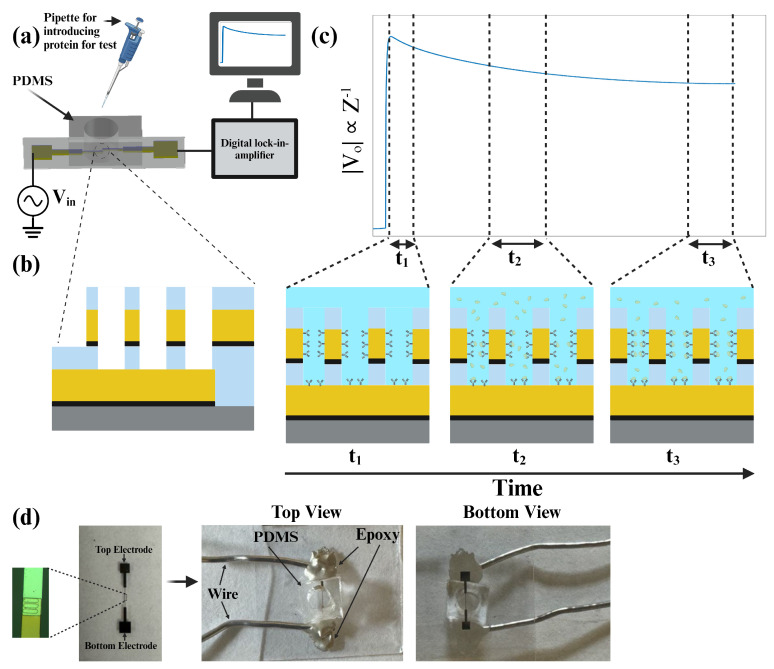
(**a**) Schematic of the designed and fabricated biosensor and experiment setup; (**b**) cross-sectional view of the electrodes and nanowells; (**c**) voltage (inversely proportional to the impedance) changes in different stages of antigen detection. More specifically, when adding antigens, the voltage decreases gradually because of the binding of antigens to the preabsorbed antibodies ($t_{1}$). After several minutes, it continues decreasing since proteins are bound to the antigens one by one but there are still more unbound antibodies on the electrode’s surface ($t_{2}$). It decreases until all possible antigens attach to the antibodies and then becomes constant ($t_{3}$); (**d**) from left to right; an image of a sensor with the zoomed-in microscopic image of the overlapping area with nanowells, a sensor bonded with PDMS channel and wires by epoxy, and an image of the sensor from the bottom of the glass wafer.

Despite the acknowledged potential of nanowell-based biosensors, there remains a notable gap in research regarding how variations in nanowell geometry influence biosensor performance. This gap underscores the necessity for studies exploring alternative nanowell structures that could improve both sensitivity and fabrication efficiency. Furthermore, previous configurations [18,19,20] face significant challenges due to the limitations of photolithography, particularly with respect to size constraints. For instance, arrays of nanowells with a diameter of 2 μm present considerable difficulties during the photolithography process. In response to this gap, our study proposes and evaluates three novel nanowell geometries—tube, spiral, and quatrefoil—designed to increase the surface area of the wells while maintaining a consistent circumference. By systematically comparing the performance of these geometries in detecting the IL-6 protein, we aim to identify an optimal nanowell design that enhances both the sensitivity and fabrication efficiency of impedance-based label-free biosensors. Figure 1 presents the sensor schematic, the protein detection test mechanism, and the sensor image. In Section 2, the fabrication process of the biosensors will be discussed in detail. Also, different characterizations of sensors will be presented. In Section 3, the results of the protein and antibody tests will be shown and compared with each other. Finally, in the conclusion section of the paper, we summarize our findings and discuss potential implications for future studies with the proposed structure of the impedance-based label-free biosensor.

### Nanowell Design and Proposed Structures

**Figure 2:**
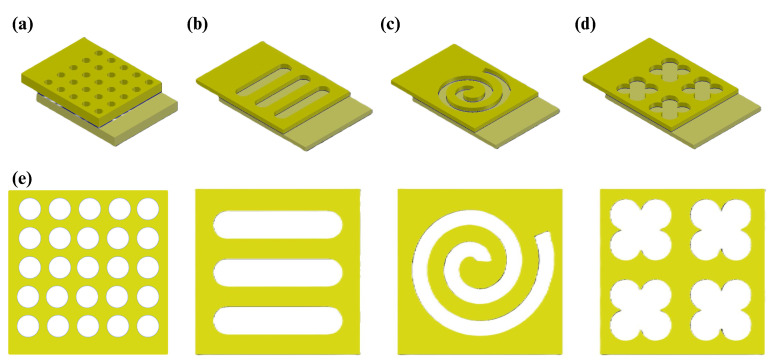
(**a**) A 3D schematic of nanowell arrays used in prior studies including an array of 5 by 5 circle-shaped nanowells with a diameter of 2 μm that makes the circumference of the wells to be around 160 μm. In this geometry, unlike the other proposed geometries, the overlapping area is a 20 by 20 μm^2^ square. (**b**) A 3D schematic of tube-shaped nanowells including three parallel tubes with a length of 27.5 μm and diameter of 7.5 μm with a total perimeter of 235 μm. (**c**) A 3D schematic of spiral-shaped nanowells including a two-round spiral with inner diameters of 1 and 9 μm and outer diameters of 36 and 44 μm with a total perimeter of 163 μm. (**d**) A 3D schematic of quatrefoil-shaped nanowells including four quatrefoils with a radius of 4.2 μm with a total perimeter of 287 μm. (**e**) A 2D top view of nanowells in the overlapping area.

As mentioned before, the structures used for previous studies were different arrays of circle-shaped wells (Figure 2a). In this paper, we tried to find other possible designs for wells, including tube, spiral, and quatrefoil, which are shown in Figure 2b, Figure 2c, and Figure 2d, respectively. Besides the well structures, the overlapping area of the electrodes was changed from a 20 by 20 μm^2^ to a 45 by 45 μm^2^ square. The fabrication process and its considerations are discussed in the next section.

The bottom electrode surface area of each well structure (considering no defection during the fabrication process) can be measured. Due to the values presented in Figure 1, the bottom electrode surface area of the tube, spiral, quatrefoil, and circle nanowells are 250, 565, 520, and 79 μm^2^, respectively. In addition, referring to Section 2.1, well heights tend to be around 185 nm. So, the effective volumes of the nanowells for protein testing are 46.25, 104.53, 96.2, and 14.62 μm^3^ for tube, spiral, quatrefoil, and circle, respectively. Consequently, we calculated the perimeter-to-area ratio of each nanowell structure, which were 0.94, 0.25, 0.55, and 2 μm^−1^ for tube, spiral, quatrefoil, and circle, respectively.

## 2. Materials and Methods

### 2.1. Microfabrication of the Biosensor

**Figure 3:**
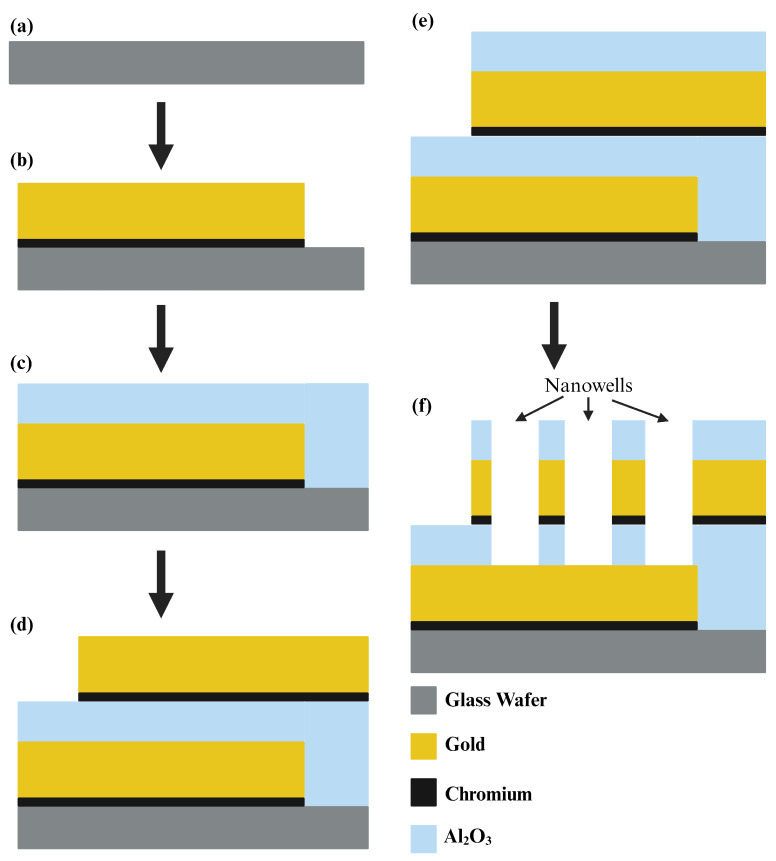
Fabrication process of the biosensor: (**a**) preparation of a glass wafer; (**b**) photolithography and metal (Cr and Au) deposition of the first electrode. In this step, a 5 nm layer of chromium as well as a 100 nm layer of gold were deposited using an electron beam deposition; (**c**) deposition of the oxide layer (Al_2_O_3_) by atomic layer deposition (ALD), which served as an insulation layer; (**d**) photolithography and metal deposition of the second electrode as well as the first electrode. There was a 45 μm by 45 μm overlapping region between two electrodes; (**e**) deposition of the oxide layer (Al_2_O_3_) by atomic layer deposition, which served as a protection and insulation layer; (**f**) wet-etching included the following four steps: (1) buffered oxide etch for etching the oxide layer; (2) Au etch for etching the gold layer; (3) Cr etch for etching the chromium layer; (4) buffered oxide etch for etching the oxide layer.

**Figure 4:**
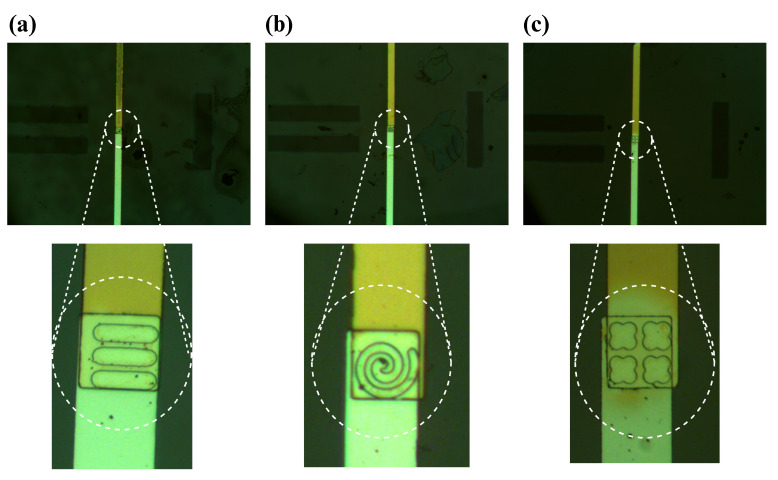
Microscopic image of (**a**) tube-shaped, (**b**) spiral-shaped, and (**c**) quatrefoil-shaped nanowells.

We utilized a 500 μm thick glass wafer, 76.2 mm in diameter, sourced from University Wafer Inc. (South Boston, MA, USA), as the foundation for our sensor. Employing optical lithography (SUSS MicroTec ReMan GmbH, Oberschleissheim, Germany), we patterned the first electrode on the wafer. For this process, AZ5214 served as the Photoresist, while AZ 917MIF acted as the developer. Subsequently, utilizing the E-beam with liquid N_2,_ we deposited a thin 5 nm layer of Cr followed by a 100 nm layer of Au onto the wafer, forming the first electrode. The lift-off process with acetone removed any unwanted deposited metals, leaving only our electrode intact. In the subsequent stage, we deposited a 40 nm layer of Al_2_O_3_ using atomic layer deposition to insulate between the two electrodes. With the fabrication of the first electrode complete, we proceeded to pattern the second electrode using the same methodology, ensuring a 45 by 45 μm^2^ overlapping area between the two electrodes. Again, we deposited a 5 nm layer of Cr followed by a 100 nm layer of Au using an E-beam with liquid N_2_ and performed a lift-off with acetone to eliminate undesired metals. Following this, another 40 nm layer of Al_2_O_3_ was deposited through atomic layer deposition to protect the electrode surface and provide insulation, preventing the sensor from capturing extraneous signals. In the final step, wells were patterned within the overlapping area of electrodes using the same procedure as for electrode patterning. Wet-etching, employing buffered oxide etch (BOE), Au, and Cr etchants, was then performed to create nanowells. Acetone was subsequently introduced to remove the residual photoresist from the sensor and wafer surface. Figure 3 illustrates the entire microfabrication process of the sensor, while microscopic images of the formed wells are depicted in Figure 4.

After fabricating sensors, we used PDMS provided by SYLGRADTM 184 Silicone Elastomer for the channel. PDMS was attached to the surface of the biosensor with an oxygen plasma cleaner (Harrick Plasma PDC001HP, Ithaca, NY, USA) which facilitates the bonding of the glass wafer to the PDMS channel. Finally, two conductive wires were bound to the electrodes by conductive epoxy (Chemtronics CW2400) to make contact between the electrodes and the lock-in-amplifier (Zurich Instruments HF2IS, Zurich, Switzerland) as a measurement device.

### 2.2. Scanning Electron Microscopy (SEM)

**Figure 5:**
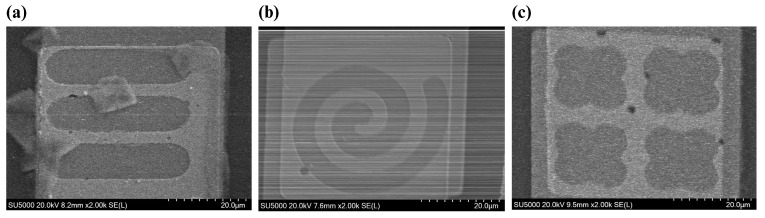
SEM images of (**a**) tube-shaped, (**b**) spiral-shaped, and (**c**) quatrefoil-shaped nanowells. As can be seen in SEM images, wet-etching was performed properly, and wells were formed. Darker areas represent the bottom electrode, while the brighter areas show the top electrode. So, inside the wells is darker, and other areas of the overlapping region are brighter.

To validate our fabrication process, scanning electron microscopy (SEM) was used with a Hitachi SU5000 Schottky Field-Emission SEM device (Hitachi High-Technologies Corporation, Tokyo, Japan). For our application, the accelerating voltage ($V_{\mathrm{acc}}$) and magnification were set to 20 KV and ×2.00 K, respectively. Figure 5 shows the formed nanowells on the electrode. SEM images verify that metal deposition and etching wells were performed properly.

### 2.3. Impedance Measurement

**Figure 6:**
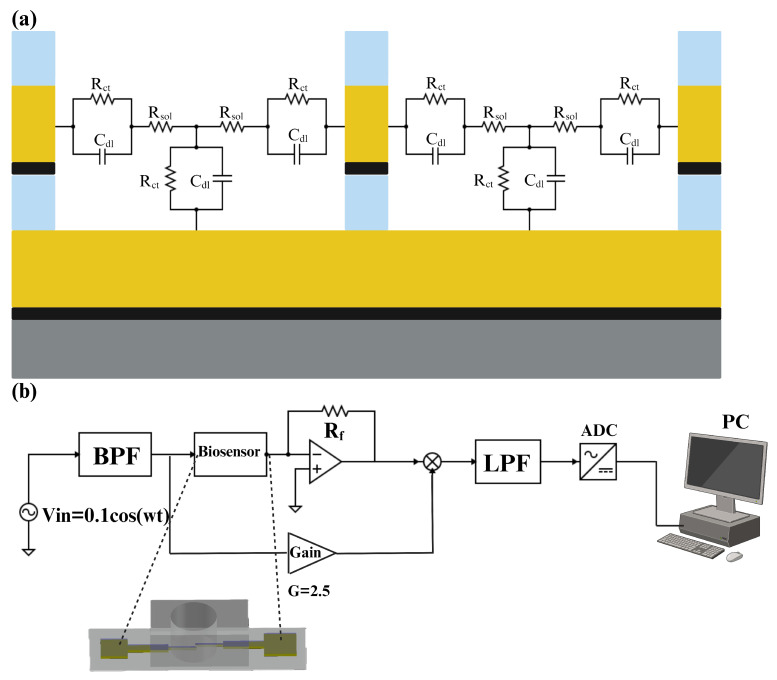
(**a**) The equivalent circuit of the electrode–electrolyte interface includes a resistor and a capacitor. These elements represent the double-layer capacitance ($C_{dl}$) and the charge transfer resistance ($R_{ct}$) at the interface between the electrodes and the solution. Additionally, there is a resistor ($R_{sol}$) that models the resistance of the solution. (**b**) The input signal was applied to one electrode of the biosensor and the captured voltage from the other electrode went through an operational amplifier with a feedback resistor of $R_{f}$. It then mixed with the amplified input signal and went through an LPF to remove the noises. Then, an ADC converted the analog signal to digital for post-processing in a PC.

The equivalent electrical circuit of an electrode–electrolyte interface is a crucial analytical tool in electrochemistry, used to model and understand the complex interactions occurring at this boundary. By representing the interface with components such as resistors, capacitors, and sometimes inductors, it becomes possible to simplify and analyze the various electrochemical phenomena [21]. The simplified equivalent electrical circuit of the electrode–electrolyte interface is represented in Figure 6a. This equivalent circuit includes one capacitor in parallel with a resistor in the contact region of the electrode and electrolyte. Double-layer capacitance refers to the capacitance that arises at the interface between an electrode and an electrolyte, where a structured layer of charged particles forms. This double layer consists of two parts: the Helmholtz layer (or Stern layer), which is directly adjacent to the electrode surface and contains specifically adsorbed ions, and the diffuse layer, which contains a distribution of ions extending into the bulk of the electrolyte [22]. Moreover, the charge transfer resistor is a component that represents the resistance to the transfer of electrons between the electrode and the electrolyte [23]. In a series with these components, there is a resistor that represents the resistance of the solution. It can be determined by the spreading resistance, which is the resistance encountered by the current as it disperses into the solution [24].

For impedance measurements, we employed a multi-frequency lock-in amplifier (Zurich Instruments HF2IS, Zurich, Switzerland). As can be seen in Figure 6b, at first, a crystal oscillator generated a 1 MHz excitation signal for the biosensor, which was then routed through an active bandpass filter to produce a 100 mV sinusoidal signal. This signal was fed through the biosensor and into the mixer, which means $V_{{electrode}_{1}}=V_{in}=0.1\cos\left(\omega t\right)$. The mixer combined the amplified signal with the biosensor’s output (after passing through an operational amplifier), and the resulting mixed signal was processed through an active low-pass filter to minimize the noise of the signal. Finally, an AC-to-DC converter was used to convert the analog data to digital data and transfer them to a PC. The choice of a 1 MHz frequency was deliberate. It strikes a balance between various factors crucial for accurate measurements. At this frequency, the equivalent impedance of the electrode–analyte interface tends toward a purely resistive nature, especially considering frequencies above 100 KHz where the double-layer capacitance effectively shorts out and there is only one resistor ($R_{sol}$) in the equivalent circuit. Importantly, this frequency was carefully selected to avoid potential damage to the gold electrodes, ensuring they were not overheated or damaged. Basically, in this research, the changes that occurred in the $R_{sol}$ would be the criteria for detecting the IL-6 antigen. However, in the Results and Discussion section, we present the change in impedance in terms of the post-processed voltage in the lock-in amplifier’s circuit, which is shown in Figure 6b as $V_{o}$. The relationship between $V_{o}$ and $R_{sol}$ can be derived by applying a simple KCL in the circuit. Equation (1) shows $V_{o}$ in terms of $R_{sol}$ and $R_{f}$

(1) $$\begin{array}{c} |V_{o}|=\frac{R_{f}}{R_{sol}}\left|V_{in}\right|. \end{array}$$

In Equation (1), $R_{f}$ and $V_{in}$ are constant parameters, so $V_{o}$ is inversely proportional to the $R_{sol}$. In this research, all plots and numbers are in terms of voltage, which is $V_{o}$ and is a coefficient of the inverse resistance of the solution.

### 2.4. Preparing Antibody and Antigen (Protein)

In our experiment, Monoclonal Human/Primate IL-6 Antibody (MAB206, R&D Systems) and Recombinant Human IL-6 Protein (206-IL-010, R&D Systems) were used as a target macromolecule to be detected by the designed and fabricated sensor. IL-6 antibodies and proteins were diluted in 1× PBS (1× phosphate-buffered saline with 7.4 pH) to reach 100 nm concentration. Notably, 1× PBS is a buffer solution commonly used in biological research containing 137 mm NaCl, 2.7 mm KCl, 10 mm Na_2_HPO_4_, and 1.8 mm KH_2_PO_4_. PBS has good electrical conductivity due to the presence of ions like Na^+^, K^+^, and Cl^+^. This is important for impedance-based experiments, as it ensures consistent and reliable measurements by providing a stable ionic environment. Plus, PBS has an ionic composition similar to that of human extracellular fluids, which makes it a suitable medium for experiments involving antibodies and proteins. Moreover, the selection of a 100 nm concentration for the sensing experiments was based on prior research and the performance metrics observed during preliminary tests. This concentration was chosen to achieve an optimal balance between detectability and sensitivity, providing a robust signal while remaining within a biologically relevant range for IL-6 detection.

## 3. Results and Discussion

**Figure 7:**
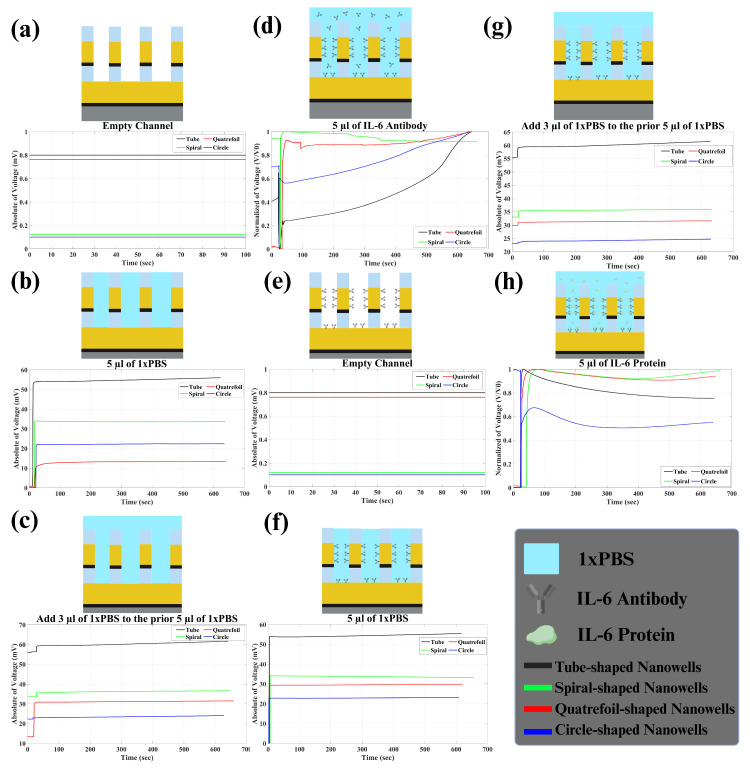
Steps of protein detection: (**a**) initially, the channel was empty, resulting in a captured voltage of hundreds of μV (around zero), indicative of an open circuit with no electrical path between the two electrodes; (**b**) adding 5 μL of PBS. In this step, an electrical path (mostly resistive) was formed through two electrodes. So, we expected to see a huge change in voltage to tens of mV; (**c**) adding more 1× PBS only slightly affected the captured voltage. Since the electrical path was already established, further addition of PBS caused a minimal change in impedance; (**d**) introducing the IL-6 antibody led to an exponential change in captured voltage until all IL-6 antibodies bound to the electrode surface, reaching saturation; (**e**) removing the solution from the channel. Note that, by removing the solution from the channel, absorbed antibodies were still attached to the surface of the electrodes. Again, there was no electrical path between the two electrodes, so the captured voltage was around zero (open circuit); (**f**) adding 5 μL of 1× PBS to the channel, now facilitated by the IL-6 antibody, altered the voltage (impedance) similarly to step (**b**); (**g**) an additional 3 μL of 1× PBS continued to have a minimal effect on voltage (impedance), consistent with the stage (**c**); (**h**) adding 5 μL of 100 nm of IL-6 protein; finally, in the last step, adding proteins caused an exponential change in impedance until all absorbed antibodies bound to the protein and then impedance became constant.

In the Results section, we detail our findings in terms of voltage variations rather than impedance (described in Section 2.3). It is important to note that changes in captured voltage exhibit an inverse relationship with impedance alterations. Our analysis involved recording impedance changes across six stages, as depicted in Figure 7. Initially, 5 μL of 1× PBS was introduced into the channel, and impedance fluctuations were monitored for ten minutes in real time. Upon the addition of 1× PBS, a resistive circuit formed, leading to a voltage shift at the second electrode and consequently altering impedance. Notably, prior to the introduction of PBS, the absence of a connection between electrodes resulted in infinite impedance (Figure 7a). Following the introduction of PBS, impedance between electrodes and solution emerged, as illustrated in Figure 7b. This stabilized impedance post-PBS addition served as our baseline. Subsequently, an additional 3 μL of PBS was introduced, resulting in minimal impedance changes (Figure 7c). Moving forward, the introduction of 5 μL of pre-prepared antibody (referenced in Section 2.4) induced immediate voltage distortion, followed by an exponential change in the captured voltage (Figure 7d). This decrease signified the gradual attachment and bonding of antibodies to the electrode surface. As antibodies bonded, impedance stabilized, suggesting saturation of electrode surfaces. This process of antibody adsorption primed the sensor for IL-6 antigen capture and detection in the subsequent step. Following the removal of the solution within the channel, the sensor was poised for protein detection testing (Figure 7e).

Once the sensor underwent pre-treatment with the specific IL-6 antibody, we proceeded with the experimentation involving IL-6 proteins. The protocol mirrored the steps taken with antibodies. Initially, 5 μL of 1× PBS was introduced into the channel, and impedance changes were monitored over 10 min, as represented in Figure 7f. We anticipated observing similar trends to those seen with antibodies. Subsequently, an additional 3 μL of 1× PBS was introduced into the channel (Figure 7g). Finally, 5 μL of IL-6 protein was introduced into the solution, prompting an exponential decrease in voltage as the proteins bound to the antibodies. Figure 7h shows the normalized voltage. This process (change in impedance) continued until all proteins were bound (it took less than ten minutes for us to monitor impedance change). We repeated these steps for the four different nanowell structures, and the corresponding data are presented in Figure 7. To effectively assess sensor sensitivity to PBS and protein, we analyzed two key metrics: baseline response and impedance variation upon protein introduction. The baseline response involved the sensor’s reaction to PBS upon introduction into an empty channel, while impedance change over time indicated the sensor’s responsiveness to the presence of protein in the channel. A stronger response to PBS and greater impedance change signified heightened sensitivity in detecting IL-6 protein, thus indicating superior sensor performance. Table 1 includes all of these parameters for each sensor.

As a result, it can clearly be concluded that tube-shaped wells showed higher sensitivity in detecting IL-6 protein. The impedance change percentage for IL-6 protein in tube nanowells was 9.55% which was higher than the ones seen for spiral, quatrefoil, and circle nanowells, which were 0.91, 0.95, and 1.62%, respectively. Also, the baselines were found to be higher for tube nanowells in comparison to the other three geometries. The first baseline (biosensor’s response to the first 5 μL of PBS when added to the empty channel) was around 56.00 mV for tube nanowells. This value tended to be higher rather than the first baseline for other geometries, which were 33.61, 13.36, and 22.32 mV, respectively. Moreover, the same thing was observed for other baselines, indicating higher sensitivity for tube nanowells. The exact values of baselines for all four geometries are presented in Table 1.

The reason for the superior sensitivity of tube-shaped wells compared to other structures remains a topic of debate. However, this enhanced sensitivity could be attributed to the increased surface area offered by tube-shaped wells relative to other geometries, such as spirals, quatrefoils, and circles. The larger surface area allows for greater interaction between biomolecules (like IL-6 antibodies and antigens) and the electrode, resulting in higher sensitivity and a more pronounced impedance change. So, the better performance in comparison to circle-shaped nanowells can be justified. Additionally, the tube geometry provides an optimal perimeter-to-area ratio, which likely improves the electrical field distribution around the nanowell. This enhanced distribution increases the likelihood of biomolecule interaction with the sensor surface, thereby enhancing detection capabilities. Also, as we mentioned before in Section Nanowell Design and Proposed Structures, the perimeter-to-area ratio of tube structure tends to be higher rather than other proposed structures including spiral and quatrefoil structures (0.94 in comparison to 0.25 and 0.55).

## 4. Conclusions

In conclusion, our research underscores the substantial benefits of optimizing nanowell geometries to enhance the performance of impedance-based, label-free biosensors in detecting cancer biomarkers. By addressing the limitations inherent in the photolithography process of conventional nanowell arrays, we proposed and tested alternative geometries including tube, spiral, and quatrefoil to increase the surface area while maintaining a consistent circumference. The experimental results, obtained through impedance measurements using a lock-in amplifier, revealed that tube-shaped nanowells exhibited the highest sensitivity for detecting interleukin-6 (IL-6) proteins. The impedance change observed for the tube-shaped nanowells was 9.55%, a marked improvement over the 0.91%, 0.95%, and 1.62% changes recorded for the spiral, quatrefoil, and circle geometries, respectively. These findings suggest that tube-shaped nanowells not only enhance the sensitivity of the biosensors but also potentially streamline the fabrication process, overcoming the efficiency issues associated with traditional 2 μm diameter nanowells. Therefore, tube-shaped nanowell structures, due to their higher surface area and optimized perimeter-to-area ratio, represent a promising advancement in the design of nanowell-based biosensors, offering significant improvements in both detection accuracy and production efficiency. Future studies should continue to explore and refine these geometrical innovations to further optimize biosensor performance and broaden their applicability in clinical diagnostics.

## Figures and Tables

**Table 1:** Baselines and impedance change in different sensors. Tube-shaped nanowell sensors showed great impedance change and baselines in comparison to the geometries, namely spirals, quatrefoils, and circles.

Nanowell Structure	Empty Channel (μV)	Baseline1 (mV)	Baseline2 (mV)	Baseline3 (mV)	Baseline4 (mV)	Impedance Change for Antibody (%)	Impedance Change for Protein (%)
Tube	800	56.00	61.82	55.43	61.60	4.05	9.55
Spiral	121	33.61	36.67	33.11	35.72	1.70	0.91
Quatrefoil	762	13.36	31.53	29.84	31.64	0.15	0.95
Circle	100	22.32	24.02	23.16	24.73	0.68	1.62

## Data Availability

All data are presented in the paper.
